# The tomato borer, *Tuta absoluta*, invading the Mediterranean Basin, originates from a single introduction from Central Chile

**DOI:** 10.1038/srep08371

**Published:** 2015-02-10

**Authors:** Thomas Guillemaud, Aurélie Blin, Isabelle Le Goff, Nicolas Desneux, Maritza Reyes, Elisabeth Tabone, Anastasia Tsagkarakou, Laura Niño, Eric Lombaert

**Affiliations:** 1 INRA, UMR 1355 Institut Sophia Agrobiotech, 06903 Sophia Antipolis, France; 2 Universidad Austral de Chile, Facultad de Ciencias Agrarias. Campus Isla Teja, Valdivia, Chile; 3 INRA, Villa Thuret, 06903 Sophia Antipolis, France; 4 Hellenic Agricultural Organization, NAGREF, Plant Protection Institute of Heraklion, Laboratory of Entomology and Agricultural Zoology, 71003 Heraklion, Greece; 5 Instituto Nacional de Investigaciones Agrícolas (INIA), Centro de Investigaciones Agropecuarias del Estado Mérida. Mérida, Venezuela

## Abstract

The Lepidopteran pest of tomato, *Tuta absoluta*, is native to South America and is invasive in the Mediterranean basin. The species' routes of invasion were investigated. The genetic variability of samples collected in South America, Europe, Africa and Middle East was analyzed using microsatellite markers to infer precisely the source of the invasive populations and to test the hypothesis of a single versus multiple introductions into the old world continents. This analysis provides strong evidence that the origin of the invading populations was unique and was close to or in Chile, and probably in Central Chile near the town of Talca in the district of Maule.

The tomato borer, *Tuta absoluta* (Meyrick) (Lepidoptera: Gelechiidae), is a serious pest species native to South America that recently became a major threat to tomato production in the Mediterranean Basin and may become a problem in most countries of Africa, and Eurasia^1^,^2^. *Tuta absoluta* was first observed outside its native range in Eastern Spain in 2006, and can now be found throughout Southern Europe, North Africa and the Middle East^1^,^2^. Precise knowledge of the source population of *T. absoluta* invading the Mediterranean basin is required to help develop strategies to control this species^3^. Such knowledge is currently lacking. The tomato borer was first observed outside South America in Eastern Spain in 2006, and two years later in North Africa. This suggests a single introduction point in Southern Europe followed by a geographical expansion in Southern Europe and North Africa. However these historical records do not reveal the identity of the source population in South America. Indirect methods based on population genetics are needed to infer the invasion routes of *T. absoluta.* Here, we analyzed the genetic variability of samples collected in South America, Europe, Africa and the Middle East to infer precisely the source of the *T. absoluta* populations invading the Mediterranean Basin and to test the hypothesis of a single versus multiple introductions into the old world continents.

## Results

Many samples were not at Hardy-Weinberg equilibrium (see Supplementary Table S1). These results together with the difficulty to unambiguously score microsatellite genotypes – a well-known phenomenon in Lepidoptera^4^ – suggested the presence of frequent null alleles. Analyses were performed with and without the 5 loci (T437, T350, T378, T482, T458) that accounted for most disequilibria. The results presented here were obtained with all 12 loci that were analyzed, and those obtained with the 5 most problematic loci were qualitatively the same. Pairwise *F_ST_* values and genotypic differentiation tests (see Supplementary Table S2) showed that samples from the native area were strongly and significantly differentiated between different countries (with *F_ST_* values ranging from 0.14 (Argentina vs Chile) to 0.31 (Argentina vs Venezuela), and *p* < 10^−5^). Samples from the same country in South America were not significantly differentiated with the notable exception of Southern and Northern Chile that were differentiated from Central Chile (with *F_ST_* of 0.075 and 0.042, respectively). Most samples from the invaded areas in Africa, Europe and Asia were moderately differentiated with mean *F_ST_* values of 0.024, except the samples from South-Eastern Spain which displayed a high level of differentiation from all the other samples from the Mediterranean basin (with mean *F_ST_* values of about 0.09, Supplementary Table S2). The intra population genetic variation measured with the microsatellites was moderate both in the native and invaded areas (Supplementary Table S1). Interestingly the mean number of alleles and Nei's diversity were larger in the samples from Africa, Europe and Asia that in the samples from most of South America, with the exception of the samples from Chile.

**Figure 1:**
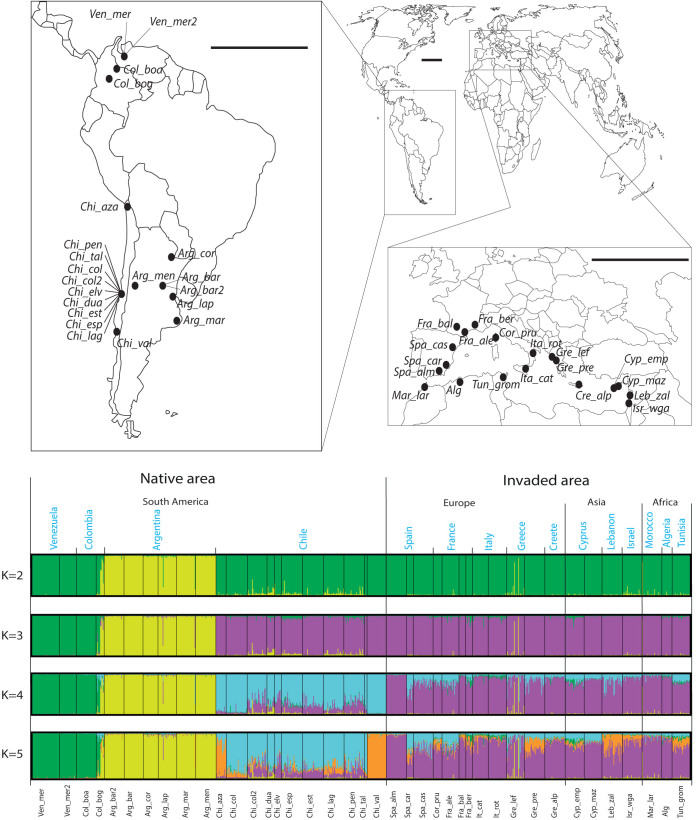
Geographic locations and Bayesian genetic clustering of the genotyped population samples of *Tuta absoluta*. Note: Bar plots of the individual genetic clustering obtained from STRUCTURE (Pritchard *et al.* 2000) are given for *K* = 2 to 5 clusters. Each vertical line represents an individual and each colour represents a genetic cluster. Individuals are grouped by population sample (names at the bottom of the figure), country, continent and type of area (names at the top of the bar plots). The map was modified from the base map freely available for non-commercial use at http://www.histgeo.ac-aix-marseille.fr. In the map, the length of the horizontal black bars is 2000 km as measured at the Earth's Equator.

**Figure 2:**
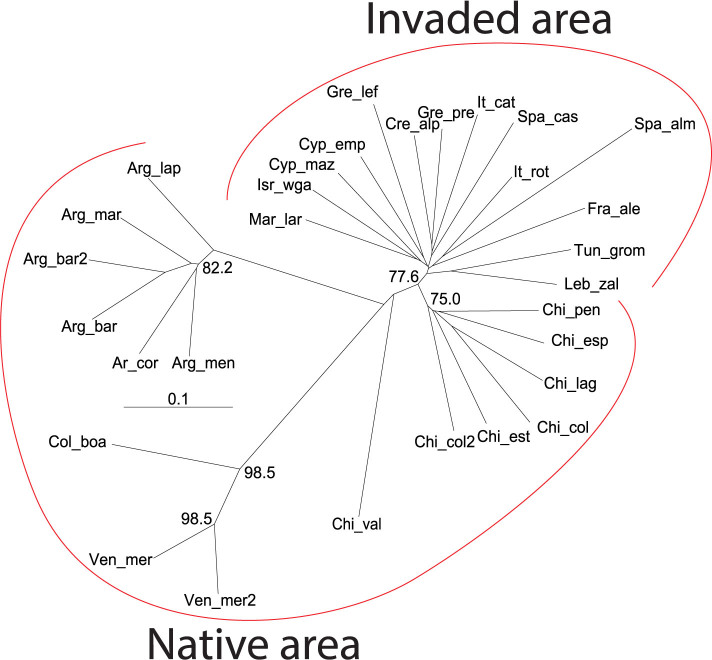
Neighbor-joining tree for the studied population samples of *Tuta absoluta* based on the distance of Cavalli-Sforza & Edwards (1967). The red curved lines group the samples according to their area of origin (indicated at the top and bottom of the tree). Bootstrap values above 50% are indicated.

**Figure 3:**
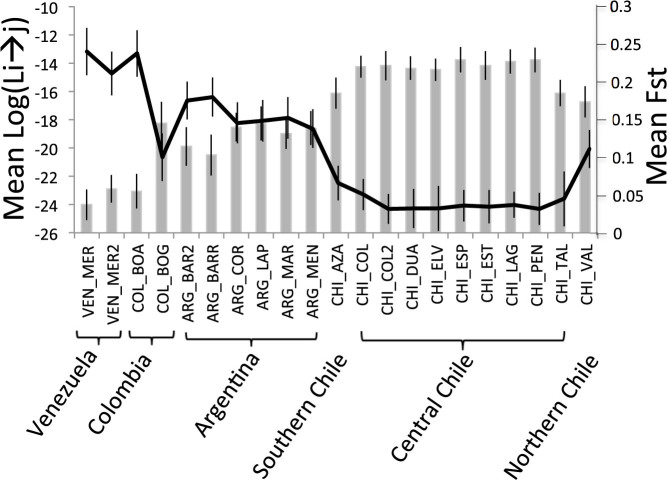
Mean pairwise FST (Weir & Cockerham, 1984) between invading population samples and each sample of the native area (line) and mean assignment likelihood of invading population samples to each sample of the native area (bar plots). The vertical thin bars are the standard deviations of the parameter considered.

Both Bayesian clustering and a Neighbor-joining (NJ) tree grouped the samples of invasive *T. absoluta* into a single cluster (Figure 1 and Figure 2). This strongly suggests a single introduction of *T. absoluta* in the Mediterranean Basin. The Bayesian clustering analysis grouped the invasive population samples together with the samples from Chile for K = 2 and K = 3 and isolated the samples from outside South America for K = 4 (Figure 1 and Supplementary Figure S1). In addition, all the samples of the invaded area displayed minimum values of pairwise *F*_ST_ with the samples from central Chile to which the maximum mean assignment likelihood, *Li*→*s*, of each African, Asian and European sample corresponded (Figure 3). Moreover the NJ tree grouped the invasive population samples together with the samples from Central Chile (Figure 2). These results all suggest that Chile, and particularly Central Chile, is the most probable source location of *T. absoluta* invading the Mediterranean Basin.

The results of the Approximate Bayesian Computation (ABC) analyses are shown in Table 1. These results clearly indicate that Chile is the true source of the population of *T. absoluta* invading the Mediterranean Basin with posterior probabilities >0.9 and non overlapping confidence intervals. Low type I and mean type II errors were obtained for both prior sets (Table 1). When Chile was removed from the analyses, the scenario with a non-sampled “ghost” South American population was selected with posterior probability >0.97 (details not shown) confirming Chile as the actual source of the invading populations of *T. absoluta*. Complementary analyses considering the North, Central and Southern part of Chile as 3 putative sources in addition to the clusters of Colombia and Argentina, and the ghost population were performed (Supplementary Figure S2). They clearly revealed that Central part of Chile is the most probable source of invasive *T. absoluta* populations (Table 1), with posterior probabilities of 0.87 and 0.67 for the first and second sets of priors and samples respectively. Again, low type I and mean type II errors were obtained for both prior sets (Table 1).

## Discussion

The main result of this study is that the origin of the invading populations around the Mediterranean was identified as unique and was in or close to Chile, and probably Central Chile in the district of Maule. Within this region, *T. absoluta* displayed very weak genetic structure so that it was not possible to infer the origin of invasive populations at a finer geographical scale. The absence of genetic structure in this region, as determined by the use of neutral genetic markers, indicates that the region surroundings Talca contains a single population of *T. absoluta*. The choice of new natural enemies (e.g. parasitoids) for possible use in biological control should take into account this new finding concerning the origin of the invasive population *T. absoluta* in the Mediterranean, as suggested by Roderick & Navajas^5^.

The second important conclusion of this study is that the native population of *T. absoluta* in South America is far from genetically homogeneous. Substantial genetic differentiation was found between northern and southern regions of South America, with more than 20% of the allele frequency variance found between southern Chile and the group of Venezuela and Colombia. Such a high level of differentiation is also found over smaller distances, for instance between Argentina and Chile or between Venezuela and Colombia. Such strong genetic structure in the native area does not usually facilitate the precise inference of the source of invasive populations because it requires an extensive sampling scheme.

The third main result was that there was an almost complete absence of genetic structuring in the invaded areas, from southern Spain to Israel and from Israel to Morocco. This genetic homogeneity over space was measured using hyper variable microsatellite markers and therefore was probably not the consequence of a low power of analysis. Instead, it very probably corresponds to a single introduction in Africa or Spain followed by an expansion without noticeable a demographic bottleneck, which would have led to genetic differentiation through space. The same situation has already been found in other recent insect invasions, such as the Asian ladybeetle *Harmonia axyridis* expansion in France^6^, the western corn rootworm *Diabrotica virgifera virgifera* expansion in North America and Central Europe^7^, and the Colorado potato beetle^8^.

## Methods

We collected samples from various regions in South America, and from the invaded area in North Africa, Europe and Asia (Figure 1, Supplementary Table S1). *T. absoluta* larvae were collected on tomato plants in greenhouses or in open field and stored in ethanol (>90%) prior to DNA extraction. Total genomic DNA of each sampled individual was extracted using the DNeasy Tissue Kit (Qiagen) following manufacturer's instructions. The genotypes of 966 individuals were obtained at 12 microsatellite markers (T454, T425, T437, T350, T235, T310, T271, T426, T478, T378, T482, T458)^9^. Intra and inter sample variability statistics, including the mean number of alleles per locus, Nei's diversity^10^, and pairwise *F*_ST_ values^11^, were computed using Genepop^12^ (ver. 4). Hardy-Weinberg (HW) and genotypic differentiation tests were performed using Fisher exact tests implemented in Genepop.

The most probable source population of the European and African invasive populations was investigated as follows^7^. 1) First, we analyzed the pair-wise *F*_ST_ values between each invasive population sample and each South American sample. 2) We then computed the mean individual assignment likelihood^13^ (denoted *Li*→*s*) of each invading population sample i, to each possible South American source population using GENECLASS2^14^ (ver. 2.0). The most probable source of a target invasive population's sample *i* was determined as the South American population whose sample displays the minimum corrected *F*_ST_ values with *i* and the maximum mean individual assignment likelihood of *i*. 3) We also plotted a neighbor joining (NJ) tree^15^ based on the genetic distance described by Cavalli-Sforza & Edwards^16^ using the POPULATIONS software version 1.2.30 (http://bioinformatics.org/~tryphon/populations/). It is expected that the source of a target invasive population's sample *i* is located in close proximity to *i* in the tree. 4) Finally, a Bayesian clustering analysis was performed using the STUCTURE software^17^ with K, the number of clusters considered varying from 1 to 10. For each value of K, an admixture model with correlated allele frequencies, the LocPrior option, 20 runs per K, 10^6^ iterations for the MCMC and 2 × 10^5^ iterations for the burn-in period were used. The most probable source of each target invasive population's sample *i* was determined as the population whose samples were the last ones to still cluster with *i* with increasing values of K. The most likely value of K was determined using the method of Evanno et al.^18^ and by eye, by examining the geographical coherence of the clustering for increasing values of K. The four above-mentioned analyses were performed on samples treated individually, i.e. without pooling samples, because frequent significant genotypic differentiation tests were found (see Supplementary Table S2).

An Approximate Bayesian Computation Analysis^19^ (ABC) was carried out with DIY ABC^20^ to measure our confidence in the source population inference. ABC is a model-based Bayesian method allowing posterior probabilities of historical scenarios to be computed, based on genetic (here, the genotypes of the samples at the 12 microsatellites) and historical data (1^st^ observation dates of the samples) and on historical and genetic parameters priors (Supplementary Table S3, Supplementary Figure S2). We contrasted 4 historical scenarios differing by the actual source population of the Mediterranean invading populations (Supplementary Figure S2). The putative source populations were the 3 South American clusters (Venezuelan/Colombian, Argentinean, and Chilean clusters) obtained from the STRUCTURE analysis described above (Figure 1) plus a non-sampled “ghost” South American population, modeling the case where the actual source population in South America had not been sampled. In each scenario the four South American populations diverged independently from an ancestral population with transitory reduction in population size at time *ti* (*i* = 1, 2, 3, 4). The analyses were conducted twice, with different parameter prior distributions and with 2 different sets of samples representative of the STRUCTURE clusters. Spa_cas and Mar_lar (see Supplementary Table S1) samples were chosen because they were large enough (>25 individuals) and they were sampled closest to the first observation point in Europe and Africa respectively^1^. The other samples representative of the STRUCTURE clusters were chosen randomly among the samples, with more than 25 individuals, belonging to each cluster.

## Author Contributions

T.G., A.T. and N.D. conceived the sampling scheme and the experiments, T.G., E.L. and A.B. analyzed the data, I.L. and A.B. performed the experiments. N.D., M.R., E.T., A.T. and L.N. organized the sampling. All authors participated to the writing of the manuscript.

## Supplementary Material

Supplementary Information Supplementary Information

## Figures and Tables

**Table 1:** Posterior probabilities of the scenarios in two sets of ABC analyses for two different sets of priors and samples. Prior and sample sets are detailed in Supplementary Table S3. 95% confidence intervals (CI) are in brackets. The 95% CI of the selected scenarios never overlapped those of competing scenarios. The values presented in italics correspond to the second set of priors and the second set of samples. Type 1 error is the probability of selecting another scenario when the chosen scenario is true. Type 2 error is the mean probability of selecting the chosen scenario when it is false. Type 2 error *i* is the probability of selecting the chosen scenario when scenario *i* is true (the mean of type 2 error *i* is type 2 error). The lines in bold characters correspond to the chosen scenarios

Set of analysis	Putative source	Prior and sample set	Posterior probability	Type 1 error	Type 2 error	Type 2 error *i*
1^st^ set	Venezuela-Colombia cluster	1	0.00 [0.00–0.00]			0.010
		2	*0.00 [0.00–0.00]*			*0.014*
	Argentina cluster	1	0.00 [0.00–0.00]			0.000
		2	*0.00 [0.00–0.00]*			*0.004*
	**Chile cluster**	**1**	**0.90 [0.87–0.94]**	**0.022**	**0.020**	-
		**2**	***0.94 [0.92–0.95]***	***0.100***	***0.035***	-
	Ghost	1	0.1 [0.06–0.13]			0.050
		2	*0.06 [0.05–0.08]*			*0.088*
2^nd^ set	Venezuela-Colombia cluster	1	0.00 [0.00–0.01]			0.004
		2	*0.00 [0.00–0.06]*			*0.004*
	Argentina cluster	1	0.00 [0.00–0.01]			0.000
		2	*0.00 [0.00–0.06]*			*0.006*
	Northern Chile	1	0.00 [0.00–0.01]			0.024
		2	*0.00 [0.00–0.06]*			*0.082*
	**Central Chile**	**1**	**0.87 [0.79–0.95]**	**0.060**	**0.015**	-
		**2**	***0.67 [0.54–0.80]***	***0.232***	***0.043***	-
	Southern Chile	1	0.13 [0.05–0.22]			0.016
		2	*0.00 [0.05–0.06]*			*0.062*
	Ghost	1	0.00 [0.00–0.00]			0.032
		2	*0.33 [0.20–0.46]*			*0.062*
