# Supplementary material for: The tomato borer, Tuta absoluta, invading the Mediterranean Basin, originates from a single introduction from Central Chile
Source: Sci Rep. 2015 Feb 10;5:8371. doi: 10.1038/srep08371 (PMC4322357; doi:10.1038/srep08371)

SUPPLEMENTARY INFORMATION

The tomato borer, *Tuta absoluta*, invading the Mediterranean Basin, originates from a single introduction from Central Chile.

Guillemaud Thomas[1], Blin Aurélie[1], Le Goff Isabelle[1], Desneux Nicolas[1], Reyes Maritza[2], Tabone Elisabeth[3], Tsagkarakou Anastasia[4], Laura Niño[5], Eric Lombaert[1]

[1] INRA, UMR 1355 Institut Sophia Agrobiotech, 06903 Sophia Antipolis, France

[2] Universidad Austral de Chile, Facultad de Ciencias Agrarias. Campus Isla Teja, Valdivia, Chile.

[3] INRA, Villa Thuret, 06903 Sophia Antipolis, France

[4] Hellenic Agricultural Organism, NAGREF, Plant Protection Institute of Heraklion, Laboratory of Entomology and Agricultural Zoology, 71003 Heraklion, Greece

[5] Instituto Nacional de Investigaciones Agrícolas (INIA), Centro de Investigaciones Agropecuarias del Estado Mérida. Mérida, Venezuela

Corresponding author: Thomas Guillemaud, guillem@sophia.inra.fr, +33 492 38 64 81 (tel), +33 492 38 64 01 (fax)

Table S1: Description of the within-population genetic variation of *Tuta absoluta* samples.

Sample	Origin			N	Sampling year	Collector	A		He	F_{IS}
	Continent	Country	Locality				DC	AR		
Ven_mer	South America	Venezuela	La Punta	40	2011	LN	4.33 (1.96)	3.52 (1.48)	0.51	-0.14*
Ven_mer2	South America	Venezuela	San Juan	25	2011	LN	4.33 (1.96)	3.67 (1.50)	0.54	-0.08
Col_boa	South America	Colombia	Boavita Boyaca	29	2011	MTL	4.25 (1.71)	3.41 (1.27)	0.51	-0.17
Col_bog	South America	Colombia	Bogota	12	2009	KW	4.33 (1.92)	-	0.64	-0.08*
Arg_bar2	South America	Argentina	Barrancas	29	2010	ML	4.58 (1.37)	4.01 (1.03)	0.59	-0.09
Arg_bar	South America	Argentina	Barrancas	28	2010	ML	4.41 (1.37)	3.73 (1.14)	0.56	-0.03
Arg_cor	South America	Argentina	Corrientes	22	2009	SC, AP	4.75 (1.42)	4.27 (1.33)	0.64	0.03
Arg_lap	South America	Argentina	La Plata	27	2010	AP	5.00 (1.65)	4.30 (1.32)	0.61	-0.17
Arg_mar	South America	Argentina	Mar Del Plata	28	2010	AP	5.00 (1.27)	4.07 (0.99)	0.59	0.02*
Arg_men	South America	Argentina	Mendoza	30	2010	AP	4.91 (1.62)	4.12 (1.25)	0.63	-0.22
Chi_aza	South America	Chile	Azapa	15	2011	MR	4.58 (1.37)	-	0.66	0.40*
Chi_col	South America	Chile	Colin	31	2011	MR	7.08 (3.31)	5.66 (2.38)	0.71	0.11*
Chi_col2	South America	Chile	Colin	29	2013	MR	7.08 (2.57)	5.62 (1.84)	0.72	0.11*
Chi_dua	South America	Chile	Duao	11	2013	MR	5.50 (1.88)	-	0.71	0.39*
Chi_elv	South America	Chile	Maule	10	2010	MR	4.66 (1.43)	-	0.72	0.02
Chi_esp	South America	Chile	Esperanza	31	2013	BL	7.41 (3.52)	5.81 (2.50)	0.72	0.03*
Chi_est	South America	Chile	Estacion Villa Alegre	31	2013	BL	6.75 (2.95)	5.51 (2.07)	0.73	0.04*
Chi_lag	South America	Chile	Lagumilla	30	2013	BL	7.25 (3.38)	5.69 (2.36)	0.70	0.12*
Chi_pen	South America	Chile	Peñaflor Nuevo	30	2013	MR	7.25 (3.19)	5.66 (2.21)	0.72	-0.28*
Chi_tal	South America	Chile	Talca	4	2013	MR	3.08 (0.9)	-	0.66	-0.06
Chi_val	South America	Chile	Valdivia	28	2011	MR	4.16 (1.58)	3.66 (1.21)	0.59	0.19*
Spa_alm	Europe	Spain	Almeria	30	2009	AU	4.50 (1.50)	4.03 (1.20)	0.60	-0.07*
Spa_car	Europe	Spain	Cartagena	10	2010	MR	3.66 (1.61)	-	0.54	-0.08
Spa_cas	Europe	Spain	Castellon	29	2009	RV	6.25 (2.63)	5.18 (1.82)	0.69	-0.02*
Cor_pru	Europe	France	Prunelli-di-Fiumorbo	13	2010	JB	5.33 (1.82)	-	0.67	0.13
Fra_ale	Europe	France	Alenya	25	2009	GR	6.00 (2.82)	5.12 (2.03)	0.68	0.06*
Fra_bal	Europe	France	Balandran	10	2010	MR	4.50 (1.67)	-	0.62	-0.17
Fra_ber	Europe	France	Berre	10	2010	MR	4.58 (1.44)	-	0.64	0.41*
It_cat	Europe	Italy	Catania	23	2009	LZ	5.33 (1.77)	4.86 (1.49)	0.69	0.26*
It_rot	Europe	Italy	Rotondella	27	2009	AS	6.50 (2.50)	5.29 (1.72)	0.71	-0.11*

Gre_lef	Europe	Greece	Lefkimmi Kerkyra	26	2009	DP	7.00 (2.21)	5.67 (1.46)	0.72	-0.12*
Gre_pre	Europe	Greece	Preveza	30	2009	DP	6.00 (1.95)	5.13 (1.67)	0.68	0.19*
Cre_alp	Europe	Greece	Ag Pelagia-Crete	30	2009	AT	6.00 (2.25)	5.00 (1.52)	0.68	0.13*
Cyp_emp	Asia	Cyprus	Empa	28	2009	VV	6.16 (1.99)	5.33 (1.63)	0.71	-0.10*
Cyp_maz	Asia	Cyprus	Mazotou	26	2010	NS	6.41 (2.23)	5.38 (1.68)	0.70	0.04*
Leb_zal	Asia	Lebanon	Zalka	30	2010	ZM	6.33 (2.57)	5.12 (1.68)	0.68	-0.11*
Isr_wga	Asia	Israël	Western Galilee	29	2011	LS	5.66 (2.06)	4.69 (1.55)	0.67	-0.27*
Mar_lar	Africa	Morocco	Larach	29	2009	AE	6.83 (2.88)	5.32 (1.65)	0.70	0.02*
Alg	Africa	Algeria	Mostaganem	15	2009	YG	5.83 (2.08)	-	0.72	-0.24
Tun_grom	Africa	Tunisia	Grombalia	26	2010	FA	6.75 (2.95)	5.66 (2.07)	0.70	-0.09*

Note: *N*: sample size. *A*: mean number of alleles per locus. *A* was determined by direct counts *(DC)* and allelic richness *(AR)* analysis. *AR* is based on sample with $N \geq 20$. Standard deviations between loci are shown in parentheses. *He*: mean expected heterozygosity. The asterisks indicate significant Hardy-Weinberg tests after sequential Bonferroni correction. "-": not applicable. Collector initials: LN: L. Niño, MTL: M. Torres-Leguizamon, KW: W. Kris, ML: M. Lieti, SC: S. Caseres, AP: A. Polack, MR: M. Reyes, BL: B. Lavandero, AU: A. Urbaneja, RV: R. Vercher, JB: J. Bodendorfer, GR: G. Ridray, LZ: L. Zappala, AS: A. Salvatore, DP: D. Papachristos, AT: A. Tsagkarakou, VV: V. Vassilou, NS: N. Seraphides, ZM: Z. Moussa, LS: L. Shaltiel, AE: A. Elamrani, YG: Y. Guenaoui, FA: F. Ajengui.

Table S2: Matrix of pair-wise F_{ST} estimates (Weir & Cockerham, 1984) of *Tuta absoluta* samples (above the diagonal) and p-values of genotypic differentiation tests (under the diagonal) computed with Genepop (Rousset, 2008). "*" indicates a significant test after sequential Bonferonni corrections.

	VEN_MER	VEN_MER	COL_BOA	COL_BOG	ARG_BAR	ARG_BAR	ARG_COR	ARG_LAP	ARG_MAR	ARG_MEN	CHI_AZA	CHI_COL	CHI_COL	CHI_DUA	CHI_ELV	CHI_ESP	CHI_EST	CHI_LAG	CHI_PEN	CHI_TAL	CHI_VAL	SPA_ALM	SPA_CAR	SPA_CAS	COR_PRU	FRA_ALE	FRA_BAL	FRA_BER	IT_CAT	IT_ROT	GRE_LEF	GRE_PRE	CRE_ALP	CYP_EMP	CYP_MAZ	LEB_ZAL	ISR_WGA	MAR_LAR	ALG	TUN_GROM
VEN_MER1		0.0048	0.1157	0.204	0.3397	0.3436	0.3207	0.3159	0.3262	0.2981	0.3041	0.2156	0.2076	0.2209	0.2527	0.203	0.2077	0.2155	0.2089	0.2319	0.2927	0.3	0.3158	0.2519	0.253	0.2222	0.2737	0.2531	0.2133	0.2246	0.2103	0.2254	0.2106	0.194	0.2491	0.2425	0.2195	0.2174	0.2289	0.233
VEN_MER	0.464		0.0981	0.1807	0.3202	0.3199	0.2967	0.2908	0.3039	0.2742	0.2637	0.183	0.1737	0.185	0.2142	0.1704	0.1737	0.1792	0.1745	0.2057	0.2703	0.2717	0.28	0.2152	0.2141	0.2034	0.2448	0.2249	0.1923	0.1947	0.1889	0.2016	0.1882	0.1661	0.2139	0.212	0.1861	0.1943	0.1977	0.2045
COL_BOA	<10-5*	<10-5*		0.1331	0.3332	0.332	0.3233	0.3001	0.3276	0.2907	0.2824	0.1988	0.2068	0.2044	0.2476	0.2042	0.2018	0.2209	0.1954	0.2639	0.2822	0.3162	0.2885	0.2391	0.2312	0.2243	0.2858	0.2609	0.2152	0.2023	0.2248	0.2276	0.2161	0.2026	0.2232	0.2265	0.2328	0.2153	0.2337	0.2241
COL_BOG	<10-5*	<10-5*	<10-5*		0.15	0.1608	0.1271	0.1205	0.1492	0.1128	0.1325	0.0663	0.0678	0.0357	0.0918	0.0649	0.0754	0.0803	0.0614	0.0905	0.1373	0.1977	0.1141	0.093	0.1027	0.0913	0.1498	0.1364	0.0873	0.0687	0.1065	0.0805	0.0901	0.0754	0.0769	0.0859	0.097	0.0765	0.0864	0.0968
ARG_BAR	<10-5*	<10-5*	<10-5*	<10-5*		-0.0021	0.0152	0.0097	0.019	0.0128	0.1744	0.1513	0.1419	0.1401	0.1467	0.1269	0.1548	0.146	0.1406	0.185	0.2117	0.2265	0.1802	0.1727	0.2021	0.1873	0.2027	0.2291	0.1789	0.1648	0.1612	0.1552	0.1664	0.1433	0.1506	0.1553	0.1671	0.147	0.175	0.1589
ARG_BAR	<10-5*	<10-5*	<10-5*	<10-5*	0.579		0.0146	0.0113	0.0117	0.0143	0.1919	0.1539	0.1395	0.1441	0.161	0.1277	0.1551	0.1435	0.1462	0.1901	0.216	0.219	0.1847	0.1803	0.2012	0.1915	0.2142	0.2397	0.1894	0.1693	0.1661	0.1616	0.1698	0.1486	0.155	0.1563	0.17	0.1449	0.188	0.1582
ARG_COR	<10-5*	<10-5*	<10-5*	<10-5*	0.004	0.003		0.0222	0.0187	0.0072	0.1562	0.1283	0.1112	0.1013	0.11	0.1028	0.1277	0.1159	0.1168	0.1476	0.1895	0.2033	0.1765	0.1393	0.1699	0.1514	0.1765	0.196	0.1487	0.1338	0.1263	0.1259	0.1329	0.1207	0.1169	0.1295	0.1342	0.1106	0.1428	0.1312
ARG_LAP	<10-5*	<10-5*	<10-5*	<10-5*	0.04	0.011	<10-5*		0.0145	0.0057	0.1696	0.1337	0.1198	0.1176	0.1306	0.107	0.1264	0.1292	0.1182	0.1504	0.1972	0.2103	0.1769	0.1386	0.1624	0.1533	0.1871	0.1993	0.1548	0.1326	0.1365	0.1349	0.1375	0.1216	0.1194	0.1272	0.1291	0.1186	0.1448	0.1325
ARG_MAR	<10-5*	<10-5*	<10-5*	<10-5*	0.018	0.052	0.005	0.017		0.0169	0.1696	0.1403	0.1242	0.1212	0.1246	0.1111	0.1409	0.1299	0.1271	0.1477	0.1942	0.2084	0.1872	0.1539	0.175	0.157	0.1925	0.1889	0.1639	0.1411	0.1354	0.1415	0.1368	0.1228	0.1222	0.1279	0.1368	0.112	0.1495	0.1336
ARG_MEN	<10-5*	<10-5*	<10-5*	<10-5*	0.005	0	0.003	0.021	0.018		0.1534	0.1126	0.1043	0.0918	0.1172	0.0947	0.1127	0.1062	0.1037	0.1275	0.19	0.1953	0.1524	0.1382	0.1569	0.1467	0.1745	0.1881	0.1431	0.1284	0.1247	0.1206	0.1263	0.1105	0.1101	0.1216	0.1266	0.1092	0.1296	0.1214
CHI_AZA	<10-5*	<10-5*	<10-5*	<10-5*	<10-5*	<10-5*	<10-5*	<10-5*	<10-5*	<10-5*		0.0466	0.0388	0.042	0.0246	0.0442	0.049	0.0332	0.0355	0.0616	0.0958	0.1206	0.1191	0.0499	0.0835	0.0735	0.0824	0.0811	0.0624	0.0577	0.0648	0.0326	0.0533	0.0432	0.0508	0.0616	0.055	0.0641	0.0508	0.0666
CHI_COL	<10-5*	<10-5*	<10-5*	<10-5*	<10-5*	<10-5*	<10-5*	<10-5*	<10-5*	<10-5*	<10-5*		0.0157	-0.011	0.0096	0.0031	0.0096	0.0031	0.0036	0.0031	0.0676	0.1092	0.087	0.0426	0.0526	0.062	0.0747	0.0647	0.0442	0.0444	0.0473	0.0312	0.0453	0.0283	0.0342	0.0445	0.0403	0.0434	0.0352	0.0512
CHI_COL	<10-5*	<10-5*	<10-5*	<10-5*	<10-5*	<10-5*	<10-5*	<10-5*	<10-5*	<10-5*	0.000001	0.000137*		0.0005	0.0164	0.0079	0.0054	-0.0042	0.0049	-0.0151	0.0714	0.0786	0.0742	0.0227	0.0262	0.0355	0.0557	0.0631	0.0278	0.0253	0.0273	0.0206	0.0156	0.0178	0.0223	0.0265	0.0227	0.023	0.0152	0.0288
CHI_DUA	<10-5*	<10-5*	<10-5*	0.002	<10-5*	<10-5*	<10-5*	<10-5*	<10-5*	<10-5*	0.000006	0.776	0.228		-0.0046	-0.0047	0.0003	-0.008	-0.0057	-0.023	0.0834	0.1046	0.0707	0.0125	0.0451	0.0425	0.0686	0.0492	0.023	0.0103	0.0212	0.0164	0.0118	0.0046	0.0177	0.038	0.0279	0.0182	0.0145	0.0389
CHI_ELV	<10-5*	<10-5*	<10-5*	<10-5*	<10-5*	<10-5*	<10-5*	<10-5*	<10-5*	<10-5*	0.009	0.241	0.093	0.573		-0.0028	0.012	0.0154	-0.0043	0.0111	0.0728	0.0973	0.1148	-0.0003	0.0419	0.0437	0.051	0.0424	0.0269	0.026	0.018	0.0199	0.0215	0.0069	0.0103	0.0312	0.0163	0.0103	0.015	0.0479
CHI_ESP	<10-5*	<10-5*	<10-5*	<10-5*	<10-5*	<10-5*	<10-5*	<10-5*	<10-5*	<10-5*	<10-5*	0.18	0.008	0.519	0.597		0.0018	0.0009	0.0012	0.0073	0.0656	0.0835	0.0841	0.0257	0.0348	0.0409	0.0556	0.0651	0.0392	0.0286	0.0314	0.0253	0.0323	0.0103	0.0185	0.0336	0.0185	0.0254	0.0223	0.0352
CHI_EST	<10-5*	<10-5*	<10-5*	<10-5*	<10-5*	<10-5*	<10-5*	<10-5*	<10-5*	<10-5*	<10-5*	0.029	0.044	0.342	0.069	0.187		0.0108	0.0048	0.0075	0.0796	0.0851	0.083	0.019	0.0238	0.0467	0.0669	0.0531	0.0248	0.0278	0.0259	0.0271	0.0337	0.0075	0.0223	0.0367	0.0177	0.0251	0.0209	0.0355
CHI_LAG	<10-5*	<10-5*	<10-5*	<10-5*	<10-5*	<10-5*	<10-5*	<10-5*	<10-5*	<10-5*	0.00001*	0.536	0.354	0.682	0.105	0.48	0.016		0.0021	-0.0071	0.0727	0.0724	0.0714	0.0395	0.0488	0.0464	0.0461	0.0683	0.0404	0.0328	0.024	0.024	0.0237	0.0147	0.0268	0.0328	0.0294	0.0332	0.0222	0.0352
CHI_PEN	<10-5*	<10-5*	<10-5*	<10-5*	<10-5*	<10-5*	<10-5*	<10-5*	<10-5*	<10-5*	0.000001	0.049	0.114	0.705	0.48	0.238	0.073	0.28		-0.0054	0.0748	0.0758	0.0837	0.0235	0.0322	0.0347	0.0534	0.0643	0.0307	0.0237	0.0248	0.0152	0.0243	0.0123	0.0147	0.0296	0.0196	0.0256	0.0092	0.0346
CHI_TAL	<10-5*	<10-5*	<10-5*	0.000216	<10-5*	<10-5*	<10-5*	<10-5*	<10-5*	<10-5*	0	0.661	0.922	0.982	0.552	0.82	0.547	0.921	0.969		0.0831	0.1346	0.1026	0.0125	0.0675	0.0438	0.1064	0.053	0.0338	0.0387	0.043	0.017	0.0037	0.0226	0.0321	0.0617	0.0397	0.0371	-0.0054	0.0337
CHI_VAL	<10-5*	<10-5*	<10-5*	<10-5*	<10-5*	<10-5*	<10-5*	<10-5*	<10-5*	<10-5*	<10-5*	<10-5*	<10-5*	<10-5*	<10-5*	<10-5*	<10-5*	<10-5*	<10-5*	<10-5*		0.1758	0.1477	0.0978	0.1131	0.1124	0.1321	0.1456	0.0981	0.105	0.1175	0.0679	0.0994	0.0832	0.098	0.0986	0.0974	0.0945	0.1186	0.1158
SPA_ALM	<10-5*	<10-5*	<10-5*	<10-5*	<10-5*	<10-5*	<10-5*	<10-5*	<10-5*	<10-5*	<10-5*	<10-5*	<10-5*	<10-5*	<10-5*	<10-5*	<10-5*	<10-5*	<10-5*	<10-5*	<10-5*		0.1513	0.0977	0.082	0.0978	0.0694	0.1602	0.0918	0.0599	0.0604	0.0972	0.099	0.0794	0.0638	0.0759	0.0776	0.0749	0.0519	0.0713
SPA_CAR	<10-5*	<10-5*	<10-5*	<10-5*	<10-5*	<10-5*	<10-5*	<10-5*	<10-5*	<10-5*	<10-5*	<10-5*	<10-5*	0.000074	<10-5*	<10-5*	<10-5*	<10-5*	<10-5*	0.006	<10-5*	<10-5*		0.109	0.1328	0.1228	0.0995	0.1609	0.0912	0.0899	0.1106	0.0675	0.0905	0.0649	0.098	0.0932	0.1174	0.0873	0.1074	0.1004
SPA_CAS	<10-5*	<10-5*	<10-5*	<10-5*	<10-5*	<10-5*	<10-5*	<10-5*	<10-5*	<10-5*	<10-5*	<10-5*	0.002	0.088	0.455	0.000044	0.000019	<10-5*	0	0.583	<10-5*	<10-5*	<10-5*		0.0175	0.027	0.0564	0.0534	0.0186	0.0112	0.0272	0.0189	0.0203	0.0208	0.0259	0.0345	0.0201	0.0178	0.0068	0.0362
COR_PRU	<10-5*	<10-5*	<10-5*	<10-5*	<10-5*	<10-5*	<10-5*	<10-5*	<10-5*	<10-5*	<10-5*	0.000042	0	0.014	0.009	0.005	0.000149	0.001	0.003	0.007	<10-5*	<10-5*	<10-5*	0.005		0.0339	0.0673	0.0704	0.0241	0.014	0.0433	0.034	0.0375	0.0349	0.0223	0.0247	0.0181	0.0129	0.0207	0.0193
FRA_ALE	<10-5*	<10-5*	<10-5*	<10-5*	<10-5*	<10-5*	<10-5*	<10-5*	<10-5*	<10-5*	<10-5*	<10-5*	0.000001	0.000033	0.000211	<10-5*	<10-5*	<10-5*	<10-5*	0.011	<10-5*	<10-5*	<10-5*	0.000165*	0		0.0679	0.0724	0.035	0.0194	0.0315	0.0202	0.0351	0.0297	0.0175	0.0387	0.0279	0.0248	0.0102	0.0255
FRA_BAL	<10-5*	<10-5*	<10-5*	<10-5*	<10-5*	<10-5*	<10-5*	<10-5*	<10-5*	<10-5*	<10-5*	<10-5*	0.000092	0.000074	0.00022*	0.000014	<10-5*	0.000021	0.000009	0.000125	<10-5*	<10-5*	<10-5*	0.001	0.000004*	0.000005*		0.1069	0.0297	0.0385	0.0268	0.0247	0.0366	0.0244	0.0476	0.0534	0.0505	0.0313	0.0312	0.0489
FRA_BER	<10-5*	<10-5*	<10-5*	<10-5*	<10-5*	<10-5*	<10-5*	<10-5*	<10-5*	<10-5*	<10-5*	<10-5*	<10-5*	0.001	0.068	0.000002	<10-5*	<10-5*	<10-5*	0.02	<10-5*	<10-5*	<10-5*	0.000026*	0.000001*	<10-5*	0.000037*		0.0486	0.0493	0.0526	0.0543	0.0402	0.0354	0.0416	0.0772	0.0354	0.0349	0.0376	0.0664
IT_CAT	<10-5*	<10-5*	<10-5*	<10-5*	<10-5*	<10-5*	<10-5*	<10-5*	<10-5*	<10-5*	<10-5*	<10-5*	0.000009	0.024	0.004	0.000002	0.000034	0.000001	0.000011	0.054	<10-5*	<10-5*	<10-5*	0.012	0.001	0.000023*	0.002	0.000105*		0.0121	0.0161	0.0025	0.0042	0.0079	0.02	0.0341	0.0139	0.0087	0.008	0.0386
IT_ROT	<10-5*	<10-5*	<10-5*	<10-5*	<10-5*	<10-5*	<10-5*	<10-5*	<10-5*	<10-5*	<10-5*	<10-5*	0.000001	0.095	0.005	<10-5*	<10-5*	0.000001	0.084	<10-5*	<10-5*	<10-5*	0.007	0.065	0.00015*	0.004	0	0.012		0.0166	0.0222	0.0159	0.0129	0.0021	0.0103	0.0059	0.0009	0.001	0.0141
GRE_LEF	<10-5*	<10-5*	<10-5*	<10-5*	<10-5*	<10-5*	<10-5*	<10-5*	<10-5*	<10-5*	<10-5*	<10-5*	0.000001	0.024	0.007	0.000001	<10-5*	0.000026	<10-5*	0.028	<10-5*	<10-5*	<10-5*	0.000111*	0.000006*	0.000006*	0.007	0.000026*	0.001	0.001		0.0188	0.0132	0.0074	0.0139	0.0341	0.0227	0.0105	0.0065	0.03
GRE_PRE	<10-5*	<10-5*	<10-5*	<10-5*	<10-5*	<10-5*	<10-5*	<10-5*	<10-5*	<10-5*	0.000019	0.000001	0.000043	0.091	0.048	0.000007	0.000001	0.000042	0.003	0.242	<10-5*	<10-5*	<10-5*	0.031	<10-5*	0.000053*	0.027	0.000149*	0.352	0.000123*	0.001		0.0037	0.0059	0.0173	0.0277	0.018	0.0101	0.0085	0.0219
CRE_ALP	<10-5*	<10-5*	<10-5*	<10-5*	<10-5*	<10-5*	<10-5*	<10-5*	<10-5*	<10-5*	<10-5*	<10-5*	0.002	0.087	0.032	0.000026	<10-5*	0.00006*	0.000234	0.283	<10-5*	<10-5*	<10-5*	0.031	<10-5*	0.000001*	0.022	0.001	0.261	0.001	0.005	0.469		0.0145	0.0165	0.0289	0.017	0.0063	0.0033	0.0318
CYP_EMP	<10-5*	<10-5*	<10-5*	<10-5*	<10-5*	<10-5*	<10-5*	<10-5*	<10-5*	<10-5*	<10-5*	<10-5*	0.000001	0.016	0.101	0.002	0.002	0.000122	0.000044	0.112	<10-5*	<10-5*	<10-5*	0.000151*	<10-5*	<10-5*	0.016	0.003	0.035	0.016	0.063	0.022	0.003		0.0184	0.0259	0.0066	0.0094	0.0086	0.0227
CYP_MAZ	<10-5*	<10-5*	<10-5*	<10-5*	<10-5*	<10-5*	<10-5*	<10-5*	<10-5*	<10-5*	<10-5*	0.000003	0.000233	0.017	0.374	0.017	0.000135	0.000079	0.001	0.237	<10-5*	<10-5*	<10-5*	0.001	0.022	0.004	0.002	0.003	0.026	0.377	0.006	0.021	0.059	0.008		0.0133	0.0017	0.0034	-0.0012	0.0205
LEB_ZAL	<10-5*	<10-5*	<10-5*	<10-5*	<10-5*	<10-5*	<10-5*	<10-5*	<10-5*	<10-5*	<10-5*	<10-5*	0.000001	0.000059	0.003	<10-5*	<10-5*	<10-5*	<10-5*	0.002	<10-5*	<10-5*	<10-5*	0.000023*	0.013	<10-5*	0	<10-5*	0.000089*	0.006	<10-5*	0.00003*	0.00001*	0.00004*	0.027		0.0125	0.019	0.0183	0.0012
ISR_WGA	<10-5*	<10-5*	<10-5*	<10-5*	<10-5*	<10-5*	<10-5*	<10-5*	<10-5*	<10-5*	<10-5*	<10-5*	0.000002	0.00003*	0.035	0.001	0.000003	<10-5*	0.000003	0.004	<10-5*	<10-5*	<10-5*	0	0.008	<10-5*	0.000039*	0	0.009	0.035	<10-5*	0	0.001	0.038	0.219	0.036		0.0073	0.0071	0.024
MAR_LAR	<10-5*	<10-5*	<10-5*	<10-5*	<10-5*	<10-5*	<10-5*	<10-5*	<10-5*	<10-5*	<10-5*	<10-5*	0.000027	0.009	0.296	0.000003	<10-5*	<10-5*	0.000002	0.073	<10-5*	<10-5*	<10-5*	0.005	0.021	0.000008*	0.005	0.002	0.014	0.163	0.006	0.038	0.142	0.16	0.379	0	0.072		0.0078	0.0214
ALG	<10-5*	<10-5*	<10-5*	<10-5*	<10-5*	<10-5*	<10-5*	<10-5*	<10-5*	<10-5*	<10-5*	0.000134	0.034	0.273	0.204	0.036	0.003	0.021	0.162	0.828	<10-5*	<10-5*	<10-5*	0.552	0.093	0.097	0.071	0.002	0.319	0.736	0.028	0.318	0.613	0.093	0.366	0.04	0.07	0.183		0.0076
TUN_GROM	<10-5*	<10-5*	<10-5*	<10-5*	<10-5*	<10-5*	<10-5*	<10-5*	<10-5*	<10-5*	<10-5*	<10-5*	0.000012	0.002	0.000092	<10-5*	<10-5*	<10-5*	<10-5*	0.196	<10-5*	<10-5*	<10-5*	0.000063*	0.051	0.000003*	0.003	0.000022*	0.000005*	0.032	0.000077*	0.002	0.000045*	0.002	0.021	0.338	0.000006*	0	0.228	

Table S3: Two sets of samples and prior distributions of demographic, historic and mutation parameters used in ABC analyses. Each set combines a group of samples and prior distributions.

	Set 1					Set 2				
samples										
Venezuela-Colombia cluster	Ven_mer					Col_boa				
Argentina cluster	Arg_bar2					Arg_lap				
Chile cluster	Chi_pen					Chi_col2				
Invasive cluster	Spa_cas					Mar_lar				
Demographic priors		mean	median	Q2.5%	Q97.5%		mean	median	Q2.5%	Q97.5%
NSi	U[1,000-100,000]	50500	50500	3480	97491	LU[500-10,000]	3180	2233	539	9284
T	U[70-100]	85	85	70.75	99.25	U[70-200]	135	135	73.25	196.75
ti	U[500-1,000]	750	750	512.5	987.5	U[200-500]	350	350	207.5	492.5
DBi	U[0-10]	5	5	0.25	9.75	U[2-7]	4.5	4.5	2.13	6.88
NFi	LU[10-1,000]	214.95	99.63	11.25	890.89	LU[1-500]	80	22	1	429
Mutational priors										
mean(μ)	U[10^{-4}-10^{-3}]	$5.5\ 10^{-4}$	$5.5\ 10^{-4}$	$1.22\ 10^{-4}$	$9.77\ 10^{-4}$	U[10^{-5}-10^{-3}]	$5.05\ 10^{-4}$	$5.05\ 10^{-4}$	$3.5\ 10^{-5}$	$9.75\ 10^{-4}$
μloc	Γ(mean(μ))	$5.5\ 10^{-4}$	$4\ 10^{-4}$	4.10^{-5}	$1.89\ 10^{-3}$	Γ(mean(μ))	$5.05\ 10^{-4}$	$3.5\ 10^{-4}$	$1.8\ 10^{-5}$	$1.85\ 10^{-3}$
mean(P)	U[10^{-1}-3.10^{-1}]	0.2	0.2	0.105	0.295	U[10^{-1}-3.10^{-1}]	0.2	0.2	0.105	0.295
Ploc	Γ(mean(P))	0.2	0.16	0.02	0.61	Γ(mean(P))	0.2	0.16	0.02	0.61
mean(μSNI)	LU[10^{-8}-3.10^{-5}]	$3.7\ 10^{-6}$	5.4710^{-7}	$1.2\ 10^{-8}$	$2.45\ 10^{-5}$	LU[10^{-8}-3.10^{-5}]	$3.7\ 10^{-6}$	5.4710^{-7}	$1.2\ 10^{-8}$	$2.45\ 10^{-5}$
μSNIloc	Γ(mean(μSNI))	$3.7\ 10^{-6}$	4.1210^{-7}	$5.69\ 10^{-9}$	$2.86\ 10^{-5}$	Γ(mean(μSN))	$3.7\ 10^{-6}$	4.1210^{-7}	$5.69\ 10^{-9}$	$2.86\ 10^{-5}$

Notes: Times were translated into numbers of generations running back in time from 2013 by assuming 10 generations per year (Desneux et al. 2010). NS = stable effective population size (number of diploid individuals); NF = effective number of founders during an introduction step lasting DB generation(s); t_i = introduction date of invasive populations i with bounds x_i fixed from dates of first observation. For microsatellite marker parameters, the loci were assumed to follow a generalized stepwise mutation model (Estoup et al. 2002) with two parameters: the mean mutation rate (mean μ) and the mean parameter of the geometric distribution (mean P) of the length in number of repeats of mutation events. Each locus has a possible range of 40 contiguous allelic states and is characterized by individual μ_{loc} and P_{loc} values, with μ_{loc} drawn from a Γ(mean=mean μ and shape=2) and P_{loc} drawn from a Γ(mean=mean P and shape=2) (Verdu et al. 2009). Uneven insertion/deletion events that were suspected for several of our microsatellite loci based on observed allele sizes (i.e. allele lengths were sometimes not multiple of the motif length implying that there has been insertion-deletion mutations (Pascual et al. 2007) were also simulated with a mean mutation rate μSNI (for single nucleotide instability) and μSNI_{loc} drawn for each locus from a Gamma(mean=mean μSNI and shape=2). Boundaries of distributions are in brackets. Parameters of Normal and Gamma distributions are in parentheses. All prior quantities presented were computed from 100,000 values. NA = not applicable; DV = can take different values; U = uniform distribution; LU = log-uniform distribution; Γ = gamma distribution.

Desneux, N., et al. (2010). Biological invasion of European tomato crops by *Tuta absoluta*: ecology, geographic expansion and prospects for biological control. Journal of Pest Science, 83(3), 197-215.
Estoup A, Jarne P, Cornuet JM (2002) Homoplasy and mutation model at microsatellite loci and their consequences for population genetics analysis. Molecular Ecology 11:1591-1604.
Pascual M, et al. (2007) Introduction history of *Drosophila subobscura* in the New World: a microsatellite-based survey using ABC methods. Molecular Ecology 16:3069-3083.
Verdu P, et al. (2009) Origins and genetic diversity of Pygmy hunter-gatherers from western central Africa. Current Biology 19:1-7.

Figure S1. A) Mean log likelihood of the data for increasing values of K and B) delta K of Evanno as a function of K. The first level of structure as determined by the delta K method of Evanno suggests K = 2: one cluster with only Argentinean samples and a second one with samples from all other countries (see Figure 1).

A)

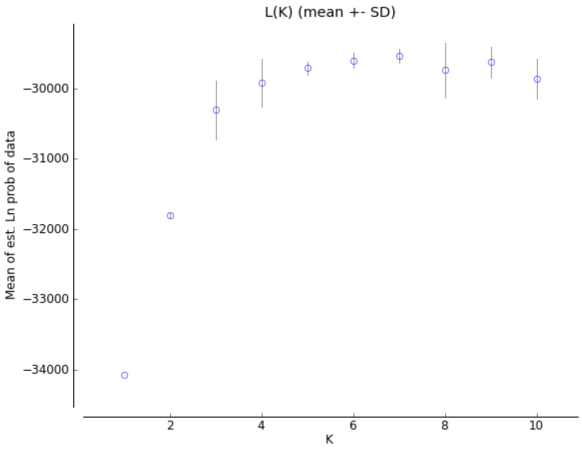

B)

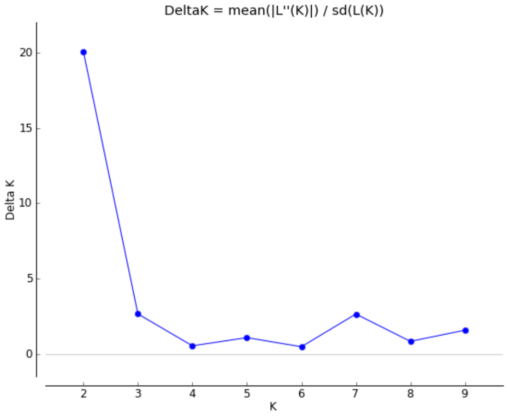

Figure S2: Graphic representation of (A) the four competing *Tuta absoluta* invasion scenarios considered in ABC analysis 1, and of (B) the six competing scenario considered in analysis 2 which focused on the origin of the invading population of the Mediterranean basin.

(A)

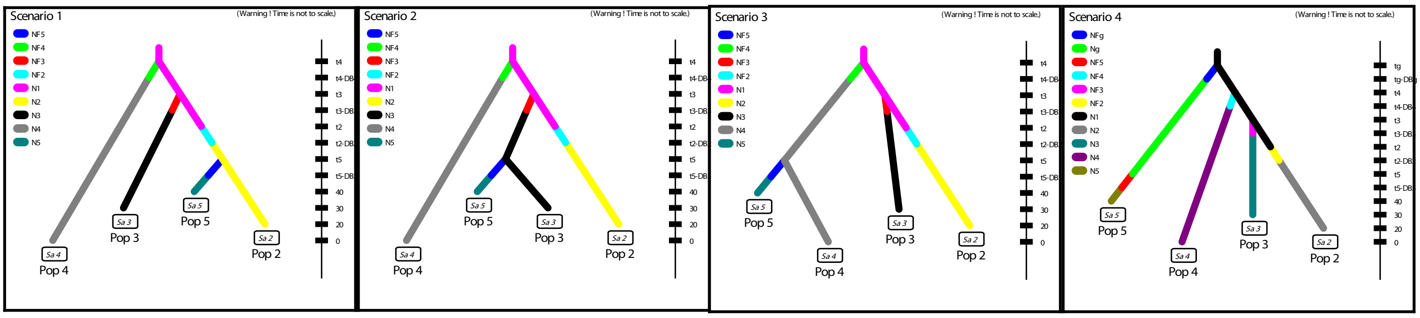

(B)

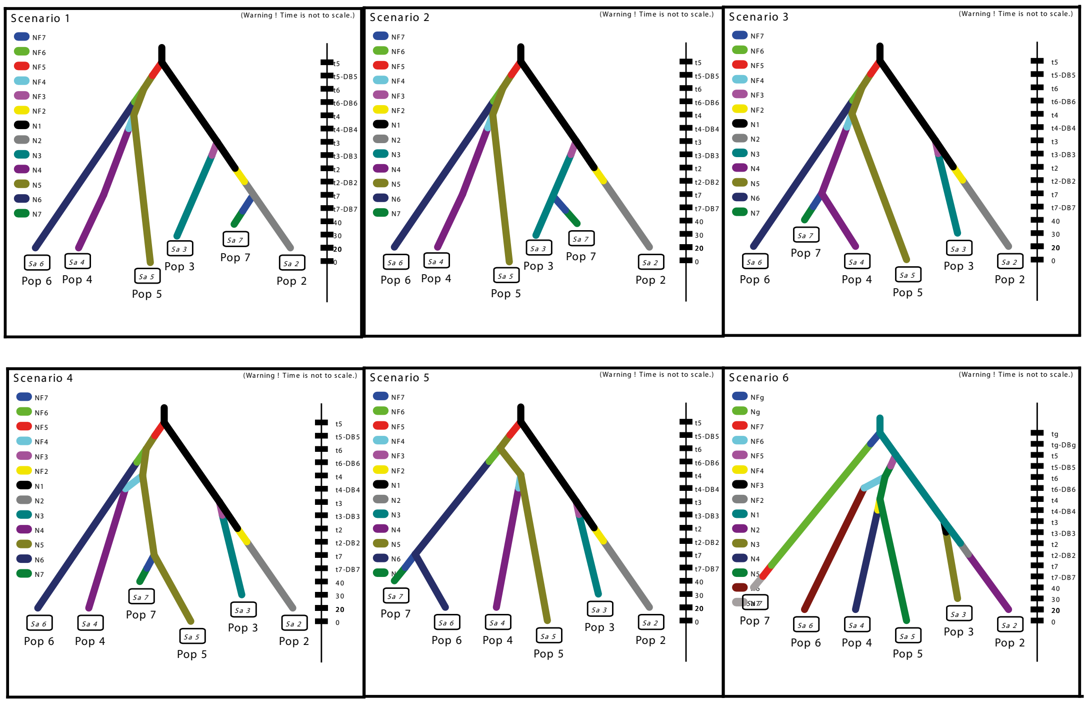

Supplement: Supplementary Information [file srep08371-s1.pdf]
